# Disposition Kinetics of Taxanes in Peritoneal Dissemination

**DOI:** 10.1155/2012/963403

**Published:** 2012-05-16

**Authors:** Ken'ichi Miyamoto, Tsutomu Shimada, Kazuki Sawamoto, Yoshimichi Sai, Yutaka Yonemura

**Affiliations:** Department of Pharmacy, Kanazawa University Hospital, 13-1 Takara-machi, Kanazawa 920-8641, Japan

## Abstract

Treatment of cancers in the abdominal cavity, such as peritoneal dissemination, is difficult, but in principle intraperitoneal administration of anticancer drugs is expected to be preferable to systemic administration. Taxane anticancer drugs are used to treat gastric cancer patients with peritoneal dissemination. They are administered as micellar preparations, Taxol and Taxotere, which consist of paclitaxel in Cremophor EL (crEL) and docetaxel in Polysorbate-80 (PS-80), respectively. In this paper we review the disposition kinetics of taxane anticancer drugs after intraperitoneal administration in peritoneal dissemination patients and animal models and also discuss the effect of the surfactant vehicle on the behavior of taxanes.

## 1. Introduction

**Figure 1:**
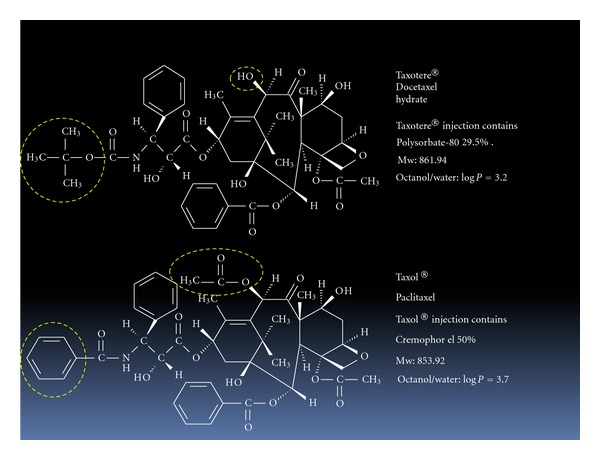
Chemical structures of taxane anticancer drugs. Circles indicate the differences between docetaxel and paclitaxel.

**Figure 2:**
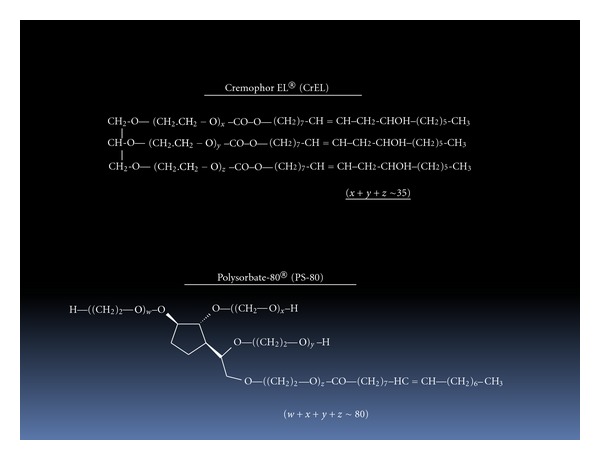
Chemical structures of the major components of Cremophor EL and Polysorbate-80.

Taxane alkaloids, paclitaxel and docetaxel, are widely used in the treatment of various cancers. Their anticancer activity is related to stabilization of microtubule assembly, and they cause mitotic arrest in the G_2_M phase of the cell cycle [1]. Paclitaxel and docetaxel have similar chemical and physical characteristics, as shown in Figure 1, and are barely soluble in various solvents. They are therefore used as micellar preparations, Taxol and Taxotere, which consist of paclitaxel in Cremophor EL (crEL) and docetaxel in Polysorbate-80 (PS-80), respectively (Figure 2).

Chemotherapy for patients with peritoneal dissemination has generally been unsatisfactory. Peritoneal cancer occurs in about 10–15% of patients with gastric cancer and in about 50–60% of relapsed cases after gastrectomy. In general, however, treatment of the peritoneal cancer is ineffective, and the 5-year survival rate is extremely low even after multidisciplinary treatment, such as surgical resection, radiotherapy, and chemotherapy. In most cases, anticancer drugs have been given by systemic administration. But, the peritoneal cavity acts as a sanctuary against systemic chemotherapy because of the existence of a blood-peritoneal barrier consisting of stromal tissue between mesothelial cells and submesothelial blood capillaries [2]. Thus, inadequate therapeutic effects might be due at least in part to failure of the drugs to reach abdominal cancerous tissues at sufficient concentration to eradicate the cancer. The intraperitoneal (i.p.) dosage route might be better than systemic administration for treatment of peritoneal dissemination, and it would be expected to produce a higher drug concentration in the abdominal cavity and to exhibit a lower systemic toxicity compared with intravenous (i.v.) administration. Fushida et al. [3, 4] and Yonemura et al. [5] tried the i.p. infusion of taxane anticancer drugs in gastric cancer patients with peritoneal dissemination and reported that the treatment was more effective, with fewer side effects, than systemic i.v. administration. Sugarbaker et al. [6] have reviewed perioperative intraperitoneal chemotherapy; they noted that the ratio of the area under the drug concentration-time curve (AUC) in the peritoneal cavity and AUC in plasma (AUC_a_/AUC_p_) was much larger for paclitaxel and docetaxel than for other anticancer drugs, suggesting that taxanes may be effective when used in early postoperative intraperitoneal chemotherapy, without severe systemic toxicity. Moreover, i.p. docetaxel appeared to be more effective than paclitaxel on peritoneal dissemination. Here, we review the disposition kinetics of taxanes after i.p. administration of taxane preparations and discuss the relationship between the pharmacokinetic characteristics and anticancer effects of taxanes, as well as the influence of the micellar surfactant vehicles.

## 2. Disposition Kinetics in Patients withPeritoneal Cancers

We investigated changes of taxane concentration in the abdominal cavity and peripheral blood after i.p. administration in advanced gastric cancer patients with peritoneal dissemination [7]. Taxol (120 mg, 180 mg) or Taxotere (60 mg, 80 mg) was dissolved in 1 L of physiological saline (final concentration of surfactant; crEL: 1.1–1.6% for Taxol, PS-80: 0.15–0.2% for Taxotere). and the preparation was infused into the peritoneal cavity of nine patients for 1 h. Blood and ascites samples were collected at designated time intervals, and the concentrations of paclitaxel and docetaxel were measured using a modification of the high-performance liquid chromatography method of Vergniol et al. [8] and Loos et al. [9].

 When Taxol (120 and 180 mg) was intraperitoneally infused at a volume of 1 L for 1 h, the maximum peritoneal concentrations of paclitaxel just after the infusion were about 110 and 190 *μ*g/mL, respectively, and decreased to 16 and 19 *μ*g/mL, respectively, after 24 h. The plasma concentration reached maximum levels of 38 and 54 ng/mL, respectively, within 3 h after the infusion and fell below the detection limit (5 ng/mL) after 24 h. On the other hand, after 1 h infusion of Taxotere (60 and 80 mg/L), the maximum peritoneal concentrations of docetaxel were 29 and 40 *μ*g/mL, respectively. These concentrations were about a half of the calculated initial concentration of docetaxel, suggesting that the drug was distributed to the peritoneal tissues or elsewhere during infusion. The peritoneal concentration was about 1 to 6 *μ*g/mL after 24 h. The plasma concentration reached the maximum levels of about 112 and 144 ng/mL, respectively, within 2 h after the infusion, then decreased to 5 to 10% of the maximum after 24 h.

Calculation of the pharmacokinetic parameters in ascitic fluid indicated that the distribution volume (Vd_a_) and the clearance (CL_a_) of docetaxel were two to three times than those of paclitaxel. Among the pharmacokinetic parameters in plasma of these drugs, Vd_p_, and CL_p_ of paclitaxel were larger than those of docetaxel, but the AUC_p_, 0−25 of docetaxel tended to be larger than that of paclitaxel. The ratio of AUC in ascitic fluid and AUC in plasma (AUC_a_/AUC_p_) was 500 to 1700 for paclitaxel and 50 to 100 for docetaxel (Table 1). Similarly, it has been reported that the AUC_a_/AUC_p_ of paclitaxel (about 1,000) [10, 11] was larger than that of docetaxel (about 200) [12, 13] after i.p. infusion. These results suggest that after infusion of taxane preparations into the peritoneal cavity, docetaxel is more easily transferred to peripheral blood vessels than paclitaxel. Namely, after i.p. infusion of Taxol the peritoneal concentration of paclitaxel was well maintained for a long time and permeation into the systemic circulation was low, suggesting that paclitaxel should be effective against peritoneal cancers, and side effects, such as bone marrow depression, should be weak. In the case of intraperitoneally administered Taxotere, the concentrations of docetaxel in the peritoneal cavity and peripheral plasma were above the cytotoxic concentration (*in vitro* IC_50_: 4–35 ng/mL) [14], so this anticancer drug may exhibit anticancer action against peritoneal cancers but may also cause systemic side effects.

## 3. Disposition Kinetics in Peritoneal Dissemination Tumor Model Animals

The rat ascites hepatoma cell line AH130 was established as transplantable tumor by Yoshida [16]. This cell line is maintained by i.p. passage at weekly intervals in female Donryu rats and is widely used to prepare animal models of peritoneal cancer dissemination. The pharmacokinetic behavior of taxane anticancer drugs and the effects of their micellar formulation vehicles have been studied using this model [15]. Four-week-old female Donryu rats were inoculated with 2 × 10^6^ AH130 cells into the peritoneal cavity and used for experiments after 1 to 2 weeks, following an overnight fast. Taxol or Taxotere was given by i.p. injection at a dose of 40 mg/kg in a 20 mL volume containing 0.2% blue dextran as a volume marker; the resulting peritoneal solutions contained 4.2% crEL for paclitacel and 1.5% PS-80 for docetaxel, which are close to the concentrations used in the case of i.v. injection of taxanes in the clinic. In the case of i.v. injection, 5 mg/kg of each drug in a volume of 200 *μ*L was administered by bolus injection into the tail vein. After i.p. or i.v. administration of taxanes to the AH130-bearing rats, the concentrations of drugs in ascitic fluid, free cancer cells, and plasma obtained from the jugular vein were measured at designated time intervals. Solid cancers in the peritoneal cavity were excised after the rats had been killed by decapitation, and the drugs were extracted and their concentrations were measured.

**Figure 3:**
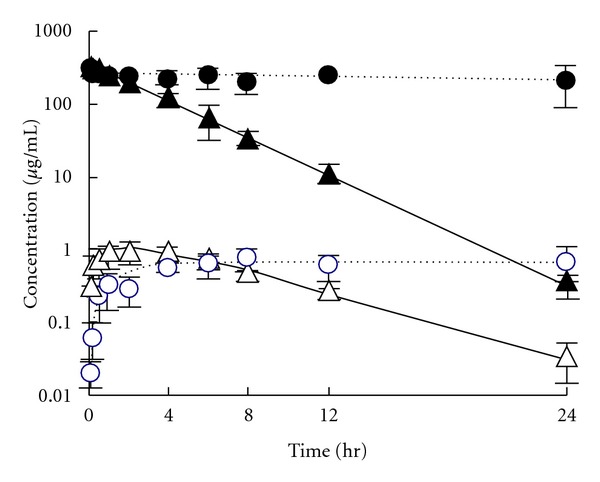
Time courses of paclitaxel (circles) or docetaxel (triangles) concentration in ascitic fluid (closed symbols) and plasma (open symbols) after an i.p. injection of 40 mg/kg of Taxol or Taxotere into AH130 tumor-bearing rats [15]. Each point with bar represents the mean ± SD of three rats.

**Figure 4:**
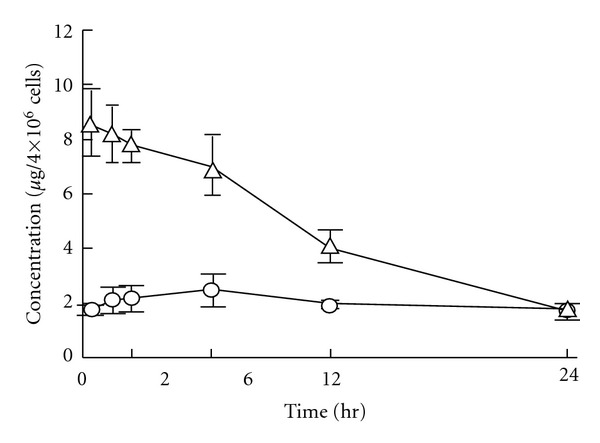
Time courses of paclitaxel (circles) or docetaxel (triangles) concentration in free tumor cells in the peritoneal cavity after an i.p. injection of 40 mg/kg of Taxol or Taxotere into AH130 tumor-bearing rats [15]. Each point with bar represents the mean ± SD of three rats.

**Figure 5:**
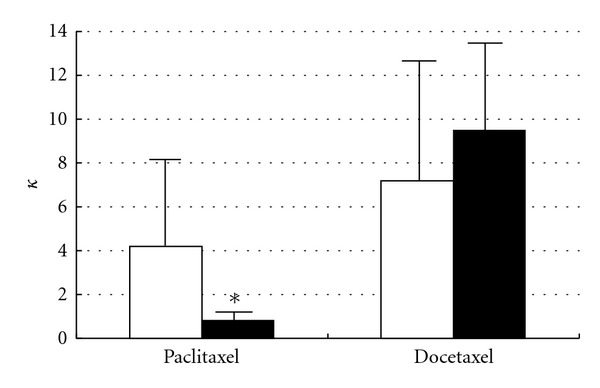
Values of apparent solid tumor to plasma concentration ratio (Kp, app) of paclitaxel and docetaxel 1 h after an i.p. (40 mg/kg, open column) or i.v. (5 mg/kg, closed column) injection of Taxol and Taxotere into AH130 tumor-bearing rats [15]. Each column with bar represents the mean ± SD of three rats. *Significantly different from docetaxel at *P* < 0.05.

 After i.p. administration of taxanes, the ascitic concentration of paclitaxel decayed very slowly, whereas that of docetaxel decreased rapidly. The plasma concentrations of both drugs were very low, but that of paclitaxel increased until 4 h and then remained at a plateau, while that of docetaxel reached the maximum at 1.5 h and then decreased (Figure 3). The values of AUC_p_, 0–24 h, and AUC_a_, 0–24 h of paclitaxel were significantly larger, by about 2- and 6-fold, respectively, than those of docetaxel, and the apparent first-order absorption rate constant from the peritoneal cavity (ka) of paclitaxel was extremely small (Table 2). The AUC_a_/AUC_p_ ratio of paclitaxel was much larger than that of docetaxel. These results indicate that paclitaxel was retained at much higher concentration than docetaxel in the peritoneal cavity after i.p. administration of taxane preparations, and the transfer of paclitaxel into the systemic circulation was much lower than that of docetaxel, in agreement with clinical findings [7, 10–13].  Figure 4 shows the changes of taxane concentration in free cancer cells in the peritoneal cavity after i.p. administration of Taxol and Taxotere (each 40 mg/kg). The concentration of paclitaxel was very low after Taxol administration, while that of docetaxel was high just after Taxotere administration and then decreased gradually in parallel with the decay of the peritoneal concentration. On the other hand, at 1 h after i.p. administration, the concentration of paclitaxel in solid cancer tissue growing in the peritoneum (1.3 ± 0.2 *μ*g/g tissue) was lower than that of docetaxel (4.1 ± 2.8 *μ*g/g tissue).  Figure 5 shows the apparent concentration ratio in solid cancer tissue versus plasma (Kp, app) 1 h after i.p. or i.v. administration. No marked difference was observed between the Kp,app values of these drugs after i.p. administration, but after i.v. administration the Kp, app of paclitaxel was significantly smaller than that of docetaxel. These results indicate that after i.p. administration of Taxol, paclitaxel was retained at high concentration in the peritoneal cavity and was not readily transferred into either the systemic circulation or cancer cells and tissues. The distribution of paclitaxel into cancer tissues was also low after i.v. administration. Docetaxel was more extensively distributed into cancer tissues than paclitaxel after administration via both routes.

**Figure 6:**
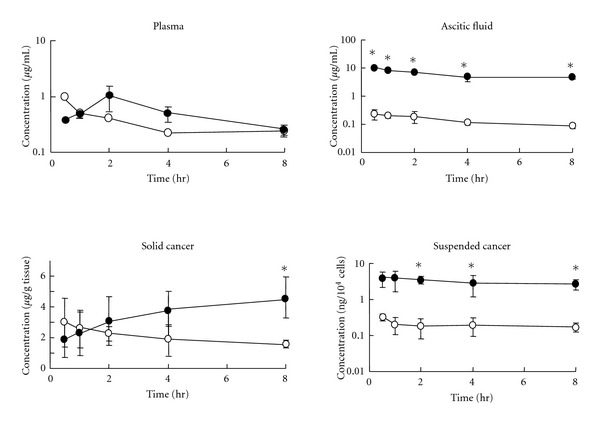
Time courses of docetaxel concentration in plasma, ascetic fluid, solid cancer, and suspended free cancer cells after an i.v. or i.p. injection of Taxotere in MKN-45P gastric cancer-bearing mice [17]. Taxotere (8 mg/kg) was i.v. (open symbols) or i.p. (closed symbols) injected into cancer-bearing mice on day 21 after i.p. inoculation of 10^7^ MKN-45P gastric cancer cells. Each point with bar represents the mean ± SD of three mice. *Significantly different from i.v. injection at *P* < 0.05.

Moreover, we found that i.p. administration of docetaxel rather than i.v. injection was pharmacokinetically superior in the treatment of peritoneal dissemination of cancer in mice [17, 19]. Docetaxel (8 mg/kg) was intravenously or intraperitoneally injected into athymic nude mice with peritoneal dissemination of MKN-45P human gastric cancer, and we measured the concentration changes in plasma, ascitic fluid, solid cancer tissue, and cancer cells suspended in the peritoneal cavity (Figure 6). The drug concentration in ascitic fluid was about 100-fold higher after i.p. injection than after i.v. injection, while the plasma concentrations were rather similar. In suspended free cancer cells in the peritoneal cavity, the drug concentration was much higher in the i.p. group than in the i.v. group, in parallel with the concentrations in ascites after drug injection via these routes. In the case of i.v. injection, the drug appeared rapidly in solid cancer tissue and then the concentration gradually decreased, following the change in the plasma concentration, but the apparent cancer tissue to plasma concentration ratio (Kp,app) was maintained at about 3 to 8 for 8 h, as observed in the AH130-bearing rat model (Figure 5). Docetaxel concentration in solid cancer was maintained at a higher level from 2 h to 8 h after i.p. injection as compared with that after i.v. injection. On the other hand, the docetaxel concentrations in normal organs rapidly decreased up to 1 h and then gradually decreased in the i.v. group, while in the i.p. group the concentrations increased up to 2 or 4 h after injection and then slowly decreased [17]. Namely, docetaxel injected into the peritoneal cavity was transferred rather slowly to the peripheral blood flow; the ratio of AUC_p_/AUC_a_ after i.p. injection of docetaxel was 0.071, but when i.v. injected, the drug passed comparatively easily into the peritoneal cavity from the blood flow; the ratio of AUC_a_/AUC_p_ after i.v. injection was 0.233 although it has been reported the existence of a blood-peritoneal barrier [2]. These results indicate that the i.p. injection of docetaxel was considered to be advantageous as a treatment method for peritoneal dissemination of cancers, offering higher local drug concentration and low systemic toxicity compared with i.v. injection.

## 4. Influence of Surfactant Vehicles on the Pharmacokinetic Behavior of Taxanes

**Figure 7:**
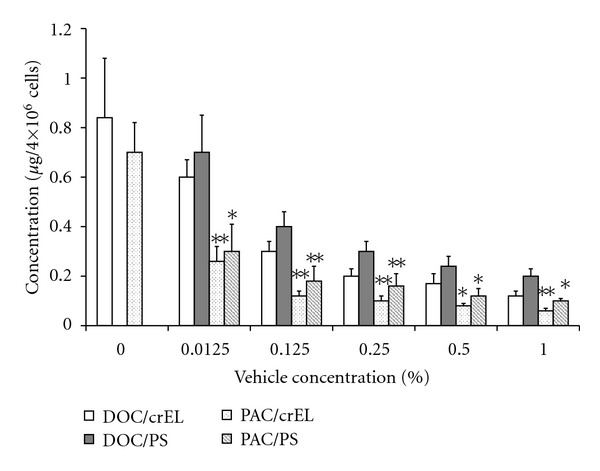
Effects of surfactants on uptake of paclitaxel and docetaxel in AH130 cells. Cells were treated with 3 *μ*g/mL of docetaxel (DOC) or paclitaxel (PAC) dissolved with 0.125% ethanol (0) or the indicated concentrations of crEL or PS-80 (PS) for 30 min. The data at 0.0125% concentration of these surfactants are taken from [15]. Each column with bar represents the mean ± SD of at least three experiments performed in triplicate. *,**Significantly different from docetaxel at *P* < 0.05 and 0.01, respectively.

Because paclitaxel and docetaxel have physicochemically similar properties, the difference of distribution after administration of these drugs may be attributed largely to the surfactant vehicles used to micellize and dissolve these drugs, but not the properties of the drugs themselves. Taxane anticancer drugs are commercially available as micellar preparations, Taxol and Taxotere, which consist of paclitaxel in crEL and docetaxel in PS-80, respectively. It has been reported that surfactants increase cellular accumulation of anticancer drugs and modulate the drug resistance of cancers expressing P-glycoprotein [20, 21]. On the other hand, crEL has been reported to inhibit the intestinal absorption and tissue permeability of paclitaxel [22–25]. However, P-glycoprotein is an efflux transporter in both multidrug-resistant cells and small intestinal epithelium cells, and therefore if these surfactants only inhibit the function of P-glycoprotein, drug accumulation should increase. This apparent contradiction may be explained as follows. CrEL increased the sensitivity of multidrug-resistant cells to daunorubicin at concentrations over 0.1 *μ*L/mL (0.01%) and completely reversed the resistance at 2.0 *μ*L/mL (0.2%) [26, 27]. PS-80 has also been shown to be a multidrug resistance modulator in vitro at concentrations between 0.2 and 0.3 *μ*L/mL (0.02–0.03%) [21, 28] but was ineffective in vivo, because of its very rapid clearance [27, 29]. Then, we examined the influence of crEL and PS-80 on the in vitro uptake of taxanes into AH130 cells, which do not express P-glycoprotein [30]. The intracellular uptake of docetaxel and paclitaxel decreased with increasing vehicle concentration (Figure 7). When these drugs were dissolved in 0.0125% ethanol (final concentration), the intracellular amounts of these drugs were similar, but in the presence of surfactants (at concentrations above 0.0125%) paclitaxel transport into the cells was less than half that of docetaxel. CrEL and PS-80 at concentrations above 0.5% both inhibited paclitaxel entry into red blood cells, in a concentration-dependent manner and with similar potency [18]. These results indicate that both surfactants inhibit the plasma membrane permeability at concentrations above 0.125%, although they can modulate the P-glycoprotein-dependent drug transport at lower concentrations. It is thought that the cell membrane permeability of taxanes is determined by the degree of affinity for, and the ease of dissociation from, surfactant micelles [31]. Paclitaxel seems to be trapped in the surfactant micelles more easily and binds to them more strongly than docetaxel.

Next, we compared the influence of surfactants on the in vivo pharmacokinetics of taxanes administered intraperitoneally to rats [18]. After injection of paclitaxel in 4.2% crEL into the peritoneal cavity, the permeation of paclitaxel into the systemic circulation was very slow compared with that of docetaxel in 1.5% PS-80. However, the permeation of docetaxel from the peritoneal cavity to the peripheral blood stream was markedly decreased by changing the surfactant from 1.5% PS-80 to 4.2% crEL though it did not reach the level of paclitaxel in 4.2% crEL. van Tellingen et al. [29] noted that PS-80 does not interfere with the disposition kinetics of docetaxel. However, the peritoneal permeability of docetaxel was lowered by increasing the concentration of PS-80 to 7.5% (Table 3).

Thus, the disposition kinetics of paclitaxel is influenced more strongly than that of docetaxel by micellar surfactants, as the concentration is increased.

## 5. Influence of Surfactants on the Anticancer Effect of Taxanes

**Figure 8:**
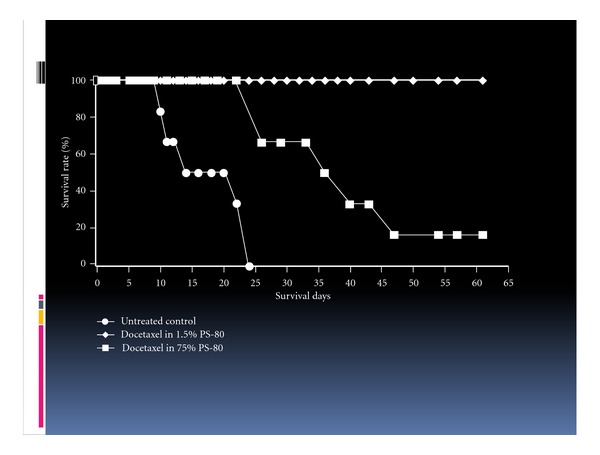
Influence of PS-80 on the anticancer effect of docetaxel (1 mg/kg) in AH130 tumor-bearing rats. AH130 tumor-bearing rats were intraperitoneally administered 1 mg/kg of docetaxel in a volume of 20 mL of 1.5% or 7.5% PS-80 on day 0. *N* = 6.

Finally, we examined the influence of surfactants on the anticancer effect of docetaxel after i.p. administration to AH130-bearing rats. The anticancer effect of docetaxel became less potent as the concentration of PS-80 was increased (Figure 8). The surfactant not only decreased the permeation of the taxane into the systemic circulation and maintained a high concentration of the drugs in the peritoneal cavity (Table 2), but also inhibited the drug transport into cancer cells, in a concentration-dependent manner, thereby reducing the anticancer effect. Similarly, it is thought that the anticancer effect of paclitaxel is strongly influenced by its vehicle, crEL, because the cell permeation of paclitaxel is readily inhibited by surfactants. The antitumor potency of Taxotere is known to be about 3 times that of Taxol. But, this difference in the potency of these antitumor drugs may be due largely to the difference in the kind and concentration of micellar surfactants used. Moreover, it has been reported that PS-80 is readily degraded by serum esterase [27, 29, 31], while crEL is stable in the body [32]. Consequently, because Taxotere readily releases docetaxel in the peritoneal cavity so that it can rapidly permeate into the systemic circulation, not only can docetaxel be directly transported into cancer cells, but also the drug can be distributed to cancer cells from the blood. This has been called the “sandwich effect” of Taxotere or the dual anticancer effect of docetaxel [33]. Taxol, a paclitaxel formulation with crEL, hardly releases the antitumor agent, so the distribution to tumors is small, and the antitumor potency may be less than that of Taxotere.

## 6. Conclusion and Perspective

Though the chemical and physical properties of taxane anticancer drugs, paclitaxel, and docetaxel are very similar, the disposition kinetics of these drugs are markedly influenced by their micellar surfactant vehicles after administration of commercial preparations. To treat peritoneal dissemination of cancers, i.p. administration seems logically preferable to systemic administration. In fact, after i.p. administration of commercial preparations diluted with physiological solution, paclitaxel showed a much higher i.p. concentration and less penetration into the systemic circulation than docetaxel. Consequently, the anticancer effect of paclitaxel appears to be stronger than that of docetaxel. However, actually the opposite is the case because the cell permeability of paclitaxel is significantly inhibited by surfactants. Taxol is a micellar formulation of paclitaxel in crEL, of which the content is much higher than in other crEL micellar preparations [34]. Taxotere is a preparation of docetaxel micellized with PS-80, which is rapidly degraded in the body and readily releases the anticancer ingredient, as compared with crEL. These characteristics seem to be the reasons why the anticancer effect of Taxotere is more potent than that of Taxol. Moreover, because many drugs are solubilized in a micellar surfactant vehicle, such as crEL, pharmacokinetic and pharmacodynamic drug-drug interactions may occur when hydrophobic drugs are administered in combination with an injection preparation containing a surfactant vehicle [35]. Further, a preparation not containing crEL is desirable to avoid hypersensitivity reaction. Recently, Abraxane has been developed as a novel crEL-free nanoparticle albumin-bound paclitaxel preparation. Data on the disposition kinetics of paclitaxel after i.p. administration of the preparation have not yet been reported and would be of considerable interest. Furthermore, hyperthermic intraperitoneal chemoperfusion (HIPEC) has been developed for treatment for peritoneal cancers with a variety of anticancer agents. It will also be important to study the pharmacokinetics of anticancer drugs in HIPEC to ensure safe and effective treatment.

## Figures and Tables

**Table 1:** The values of AUC of paclitaxel and docetaxel in plasma and ascitic fluid after an i.p. infusion of Taxol and Taxotere in patients with peritoneal tumor [7].

		AUC_p_	AUC_a_	Ratio of
		(mg∗hr/L)	(mg∗hr/L)	AUC_a_/AUC_p_
Paclitaxel	120 mg	2.57 ± 1.43	1,298 ± 238	505
	180 mg	1.30 ± 0.86	2,214 ± 128	1705

Docetaxel	60 mg	6.65 ± 3.75	370 ± 87	56
	80 mg	2.27 ± 0.65	238 ± 24	105

The value of AUC was calculated from 0 to 25 h including the period of the infusion administration.

Each value represents the mean ± SE of three patients.

*Significantly different from Taxotere at *P* < 0.01.

**Table 2:** The values of AUC of paclitaxel and docetaxel in plasma and ascitic fluid after an i.p. injection of Taxol and Taxotere into AH130 tumor-bearing rats [15].

	ka	AUC_p_	AUC_a_	Ratio of
	(hr^−1^)	(mg∗hr/L)	(mg∗hr/L)	AUC_a_/AUC_p_
Paclitaxel	0.0424 ± 0.0011*	17.6 ± 5.8*	7,480 ± 255*	425
Docetaxel	0.325 ± 0.043	8.50 ± 3.27	1,300 ± 191	153

The value of AUC was calculated from 0 to 24 h after an i.p. administration of 40 mg/kg of each drug.

ka: the apparent first-order absorption rate constant from the peritoneal cavity.

Each value represents the mean ± SD of three rats.

*Significantly different from Taxotere at *P *< 0.01.

**Table 3:** Pharmacokinetic parameters of paclitaxel (PAC) and docetaxel (DOC) in plasma and ascitic fluid after an i.p. administration of drugs in crEL or PS-80 to nontumor rats [18].

	ka	AUC_p_	AUC_a_	Ratio of
	(hr^−1^)	(mg∗hr/L)	(mg∗hr/L)	AUC_p_/AUC_a_
PAC in 4.2% crEL	0.019 ± 0.0018*	18.4 ± 3.3*	8,870 ± 790*	0.00207 ± 0.00029*
DOC in 1.5% PS-80	0.394 ± 0.021	6.93 ± 1.32	1,170 ± 120	0.00592 ± 0.00153
DOC in 4.2% crEL	0.165 ± 0.004	8.59 ± 1.23	3,520 ± 110	0.00244 ± 0.00026
DOC in 7.5% PS-80	0.130 ± 0.005	11.4 ± 1.1	3,130 ± 320	0.00364 ± 0.00026

The value of AUC was calculated from 0 to 24 h after an i.p. administration of 40 mg/kg of each drug.

ka: the apparent first-order absorption rate constant from the peritoneal cavity.

Each value represents the mean ± SD of three rats.

*Significantly different from DOC in 1.5% PS-80 at *P* < 0.01.
